# Whole home exercise intervention for depression in older care home residents (the OPERA study): a process evaluation

**DOI:** 10.1186/1741-7015-12-1

**Published:** 2014-01-03

**Authors:** David R Ellard, Margaret Thorogood, Martin Underwood, Clive Seale, Stephanie JC Taylor

**Affiliations:** 1 Warwick Clinical Trials Unit, Division of Health Sciences, Warwick Medical School, The University of Warwick, Coventry CV4 7AL, UK; 2 Division of Health Sciences, Warwick Medical School, The University of Warwick, Coventry CV4 7AL, UK; 3 Brunel University, Uxbridge, London UB8 3PH, UK; 4 Blizard Institute, Queen Mary, University of London, Barts and the London School of Medicine and Dentistry, London, UK

**Keywords:** Elderly residential care, Process evaluation, Exercise, Depression, Culture change, Cluster randomised controlled trial

## Abstract

**Background:**

The ‘Older People’s Exercise intervention in Residential and nursing Accommodation’ (OPERA) cluster randomised trial evaluated the impact of training for care home staff together with twice-weekly, physiotherapist-led exercise classes on depressive symptoms in care home residents, but found no effect. We report a process evaluation exploring potential explanations for the lack of effect.

**Methods:**

The OPERA trial included over 1,000 residents in 78 care homes in the UK. We used a mixed methods approach including quantitative data collected from all homes. In eight case study homes, we carried out repeated periods of observation and interviews with residents, care staff and managers. At the end of the intervention, we held focus groups with OPERA research staff. We reported our first findings before the trial outcome was known.

**Results:**

Homes showed large variations in activity at baseline and throughout the trial. Overall attendance rate at the group exercise sessions was low (50%). We considered two issues that might explain the negative outcome: whether the intervention changed the culture of the homes, and whether the residents engaged with the intervention. We found low levels of staff training, few home champions for the intervention and a culture that prioritised protecting residents from harm over encouraging activity. The trial team delivered 3,191 exercise groups but only 36% of participants attended at least 1 group per week and depressed residents attended significantly fewer groups than those who were not depressed. Residents were very frail and therefore most groups only included seated exercises.

**Conclusions:**

The intervention did not change the culture of the homes and, in the case study homes, activity levels did not change outside the exercise groups. Residents did not engage in the exercise groups at a sufficient level, and this was particularly true for those with depressive symptoms at baseline. The physical and mental frailty of care home residents may make it impossible to deliver a sufficiently intense exercise intervention to impact on depressive symptoms.

## Introduction

Depression is common in older people living in residential and nursing homes (care homes). Up to 40% of care home residents are depressed [1,2] and in about half of cases the depression is not resolved within a year [3]. In many cases, the care home staff or health professionals do not recognize depression [4,5]. At the conception of the OPERA trial, described below, there was sufficient evidence to support the hypothesis that increased physical activity might reduce the burden of depression in older care home residents [6].

The OPERA (‘Older People’s Exercise intervention in Residential and nursing Accommodation’) trial was a cluster randomised trial based in care homes, in which we set out to test the impact of a whole home intervention including a structured, progressive physical activity programme on the incidence and prevalence of depressive symptoms [7]. Although it was a large, adequately powered trial of a 12-month-long intervention, the OPERA trial found no detectable effect of the intervention on either the prevalence or incidence of depression [6,8]. There was also no observable effect on any of the predefined secondary outcomes.

Process evaluations may be employed early in the development of new complex interventions or as a technique to study the implementation of interventions of proven effectiveness but, as Oakley and colleagues have argued, they may also greatly improve and inform the findings of randomised controlled trials (RCTs) [9]. They outline a framework for process evaluation as an integral element of RCTs [9]. Process evaluations are increasingly being funded and embedded into the study designs of RCTs of complex interventions aiming to answer key questions about the implementation of the intervention and to place final results into context. However, there are still very few published process evaluation protocols and only a limited number of published process evaluations alongside RCTs. We carried out a process evaluation alongside the OPERA trial [10], collecting both qualitative and quantitative data on factors such as reach and uptake. In this paper, we draw on the findings of this process evaluation to explore potential explanations for the lack of effect of the intervention in the OPERA trial.

## Methods

### The randomised trial

The OPERA trial was a cluster randomised trial including 78 care homes in England; the unit of randomisation was the individual care home. Protocols with detailed descriptions of the methods and the intervention are available elsewhere [7,10]. The trial took place from 2008 to 2011 and we recruited care homes from the Midlands (Coventry and Warwickshire) and North East London, UK. The physical activity intervention involved both activities aimed at changing the culture of the home and, most importantly, twice-weekly, moderate intensity, progressive group exercise sessions led by a physiotherapist. Activities were aimed at changing the culture of the homes so that residents would be supported and encouraged to be more active. These included individual physiotherapy assessments and exercise prescriptions for all residents, advice for staff on ways to safely increase the mobility of the residents, the provision of simple aids to maximise individuals’ mobility, and formal care home staff training on recognising depression and the potential importance of promoting physical activity in residents. We planned to introduce a home ‘champion’ into each intervention home who would be a member of staff who would encourage and reinforce safe mobility and ‘activity’ when the physiotherapist was not there. Control homes only received formal staff training on depression awareness without any specific mention of promoting physical activity.

The primary outcomes of the trial were the prevalence of depression in participants able to complete assessments 12 months after randomisation, and the change in the number of depressive symptoms in all participating residents at 12 months after randomisation plus the change in number of depressive symptoms 6 months after randomisation in those participating who were depressed at baseline (based on the 15-item Geriatric Depression Scale (GDS-15) [11]). Secondary outcomes included remission of depression, cognitive function (Mini Mental State Examination (MMSE) [12]), health-related quality of life [13], mobility (Short Physical Performance Battery (SPPB) [14,15]) and exercise tolerance, pain, fear of falling, social engagement, health utility, falls and mortality rates.

### Process evaluation

We have reported full details of our methods elsewhere [10]. Briefly, we used a mixed methods approach combining quantitative data from all the study homes and quantitative and qualitative data from a purposive sample of eight case study homes (two control and six intervention) that were studied in depth. We used a sampling frame representing the different type of homes within the study (for example, size, location and ownership) to select the eight case study homes [10].

Across all the homes in the study we collected quantitative data including size, occupancy, and home facilities from the Care Quality Commission website (http://www.cqc.org.uk/cqcdata) and directly from care home managers prior to randomisation via our field researchers. We also collected trial process data from the control and intervention arms, on the staff training and, in the intervention homes only, on the delivery and receipt of the intervention. We evaluated the staff training by means of questionnaires handed out to staff at the end of each training session. They were asked to rate five statements on a five-point scale, anchored ‘strongly disagree’ (1) to ‘strongly agree’ (5). The statements were: ‘the session was relevant to my job’, ‘I learned something new from the session’, ‘I am glad I attended this session’ and ‘the session was about the right length’. In addition, there was space for comments or suggestions. We also attempted to obtain a long-term evaluation of the staff training by means of a second questionnaire mailed out to staff who had attended the training approximately 3 months after the training. This questionnaire asked staff to reflect on the training delivered and the materials that went with it and to state if they found them useful or not. Respondents were also asked to comment on the components they found most and least useful and whether they had become more aware of depression among residents since the training. Finally, they were asked if they required more information about depression, how they would like this delivered, and for any comments and suggestions. There was a slight difference in the questionnaires sent to staff in intervention homes, as this had an additional section asking about the mobility advice that was given in these homes during training.

We assessed the quality and fidelity of the intervention delivery during a site visit by the study’s lead physiotherapist to each physiotherapist involved in the delivery of the intervention at one of their assigned care homes on at least two occasions: at 6 weeks and at 6 months after the intervention started in that home. At these visits the delivery of the exercise group and use of the whole home approach were assessed by observation, and a sample of the intervention data collection and clinical record forms and registers were checked (unless any major issues were identified, in which case all were checked). An observation tool developed for this purpose was used to ensure a consistent approach. Each item was scored on a strict three-level criterion (not achieved, partially achieved, or satisfactorily achieved). Additionally, a senior researcher not involved in the delivery of the intervention (DRE) observed 21 exercise classes in the case study care homes. Sampling of classes for observation included seven in the early stages after their introduction (between weeks 3 and 5), six at about the midpoint (between weeks 24 and 27) and eight at the end of the 12 months (between weeks 45 and 48). Observation included how the session fitted into the day, how the residents were reacting, how the physiotherapist was interacting with the residents, care staff/home involvement and what happened when it ended.

In the eight case study homes the experienced qualitative researcher (DRE) conducted face to face, semistructured interviews with managers, and a sample of staff, residents and their relatives; at baseline (within the first 2 weeks) and in towards the end of the trial (between weeks 46 and 52). A small number of managers were interviewed at approximately the midpoint (between weeks 24 and 27). DRE also conducted a detailed ethnographic study in these homes observing routine daily life. We adopted a phenomenological approach for all qualitative work [16,17]. Focus groups were held with the intervention physiotherapists and the recruiting team at the end of the study to ensure their input.

Finally, we collected quantitative, descriptive data on activity within the case study homes at three timepoints (baseline, around 6 months and around 12 months) using the Behaviour Category Codes (BCC) instrument [18,19]. This involved completing a checklist of what residents were doing, including interactions with staff and others, at regular intervals in the day. Observational data sweeps occurred every 15 minutes, for a 90-minute period (that is, six sweeps in 90 minutes). Each sweep recorded the total number of residents within each public area of the home and the number of residents engaged in particular behaviours at that timepoint. DRE carried out observations starting at different times but covering a whole day over a number of visits. Behaviours were collapsed into seven key ‘activity’ behaviours for analysis (active social interaction, eating/drinking, recreational activity (not exercise), exercise, passive social interaction, socially inactive, walking/wandering). The trial was completed prior to activity data analysis to avoid influencing the data collection.

Ethical review for the trial and its process evaluation was provided by the Joint University College London/University College London Hospital Committees on the Ethics of Human Research (Committee A), now known as Central London REC 4. The REC reference for the study is 07/Q0505/56. All participants provided written informed consent to participate.

### Process data analysis

We digitally recorded interviews, subject to permission of each participant, and where appropriate, transcribed verbatim after anonymisation. Transcripts were managed using NVivo 7 QSR International Pty Ltd. Version 7, 2006. Researcher bias was minimised through regular crosschecking of data and findings by the members of research team. To ensure reliability, another member of the team (CS) coded a sample of 10% of transcripts. These two perspectives and subsequent discussions aided in the development of the coding scheme. The analysis was thematic and we adopted the framework method described by Ritchie and Spencer [20] and Pope *et al*. [21]. We use quotations as exemplars of key themes. In the quotations, an assigned code identifies the respondent, their role, and timepoint within the study of the interview (BL = baseline, FU = follow-up).

Quantitative data were analysed using the statistical package SPSS (Version 18; SPSS, Chicago, IL, USA). Analysis was largely exploratory, not hypothesis driven and necessarily unadjusted for baseline covariates. Data from the activity sweeps in the case study homes are summarised in tables (see below) with activities collapsed into groups. We based the mean percentages quoted on 12 to 14 observation sweeps of 90 minutes each covering the period from 10.30 am in the morning to 17.30 pm in each of the 8 case study homes at baseline and end of study. Change is follow-up minus baseline.

As the results were examined, a number of *post hoc* subgroup analyses were carried to looking at the effect key baseline variables (age, cognition, physical frailty and depression) on the number of groups a participant would attend. We present descriptive statistics for these including both means (SD) and medians (ranges). As these data were not normally distributed, the tests carried out were non-parametric.

## Results

### Early analysis

After the trial had closed and while the analysis of the trial results was ongoing, we carried out a first analysis of the process evaluation results that we presented to the trial management team before the trial outcomes were known [6]. At baseline, we found an enormous variation between the homes in the level of activity within them. The variation was greater than the change that it was envisaged the OPERA intervention might induce. We therefore hypothesised that the magnitude of any effect from the OPERA intervention was likely to vary considerably between homes because some homes had greater potential to increase physical activity than others, and that this variability would contribute to a small overall effect size. We also observed that attendance at the exercise classes varied considerably and that overall attendance rates were little more than 50%. We hypothesised that a dose effect would be discernible, with those residents attending a greater number of group exercise sessions showing greater beneficial effect. However, we also thought it possible that the residents without depressive symptoms might be more frequent attendees at exercise classes than those who were depressed, in which case a dose effect may be hard to detect.

### Review of findings following report of trial outcomes

After the main OPERA trial results were available, we returned to our findings to consider possible explanations for the negative result. It may be that increasing physical activity in this group is not effective in addressing depression. If so, none of our process evaluation results could add any further information. However, a second possibility is that the OPERA intervention did not actually increase physical activity as intended. This in turn could be either because the intervention was not successfully implemented or because the OPERA intervention was intrinsically ineffective at increasing physical activity. We considered these two aspects of the OPERA intervention and investigated whether the intervention changed the culture of the homes and delivered the exercise classes as intended and whether the residents attended the exercise classes as intended.

### Did the intervention change the culture in the homes?

#### Influence of physiotherapists

A key aspect of the whole home intervention was the involvement of the physiotherapists, who saw all residents and made recommendations for increasing their mobility, training staff in how to encourage mobility as well as arranging to supply mobility aids where necessary. In the post-intervention focus group, physiotherapists reported seeing that their recommendations were acted upon, encouraging walking instead of using a wheelchair, reinforcing good principles of manual handling, promoting activity and breaking down barriers to mobility. However, during observation visits DRE did not witness any episodes where additional physical activity was obviously being encouraged as a result of the OPERA intervention. For example, staff still seemed reluctant to walk with residents to mealtimes rather than to take them in a wheelchair. Managers were generally positive about the experience of having a physiotherapist working in the home and especially pleased with the supply of mobility aids (none of which were different from those that should have been available to residents as part of routine NHS provision).

It’s not just ‘Right, I want you to do this and I want you to do that’. She actually does it with them and shows them how to do it first and then they’ll try it and she’ll say ‘No, not quite like that’, you know? (1 Carer BL).

Yes, that was another asset I think that [Name] and the team brought in, which was yes, we have people walking with the walking aids and the frames and the walking sticks, but the good thing is that for me to get everybody checked here… they assessed each person… (20 Manager FU).

I’m finding it quite hard to understand why she’s put in her referrals and the equipment had come within a couple of days. If you go via the usual channels (for example, GP referrals) we’ve waited an age’ (16 Manager BL).

During a post-intervention focus group with the physiotherapists they collectively noted that, despite the intervention lasting for 12 months, there was little time to facilitate a lasting change in staff behaviour around encouraging residents’ mobility. The barriers to facilitating change included staff turnover, staff attitudes towards manual handling, staff morale and time constraints.

At times it was difficult to explain our remit to staff. We had little time to change attitudes of some staff to issues of mobility; making it hard to facilitate a change in practice. Sometimes this was due to issues of time; time to mobilise, changeover of staff, attitudes towards handling, morale and carers’ time constraints (Physiotherapist).

Expectations of what we could achieve, within a short time, were often very high perhaps giving false hope of recovery to residents and staff (Physiotherapist).

#### OPERA champions

Another part of the intended intervention was to identify champions for the OPERA changes in each intervention home. However, in some homes this did not happen at all and in others a carer took on the role but problems with shifts, time or enthusiasm limited their effectiveness. Some homes already had an ‘activities coordinator’ who took on the role of OPERA champion, but this was also not effective, either because the person did not feel this was part of their remit or the relevant person was also required for basic care duties.

#### Staff training

The trial team delivered 142 training sessions. The team in the 35 intervention homes delivered 69 sessions to 406 staff, covering just 46% of staff. Staff failed to attend because their shift arrangements were inconvenient or in a small number of cases because the information about the session was so poorly disseminated in the home that the trainer arrived to find staff unaware that the training was happening and so they were unable to attend.

At observation, we found that the training was delivered as intended in the protocol, but there were significant challenges in delivering it. Not all homes had a quiet space for staff to attend training. Attendance was unpredictable; some homes did not pay staff for time spent in training, thus reducing the incentive to attend. This was a particular disincentive for those with a day off or with a shift that started later or ended earlier than the session. In addition, on some occasions staff were called away from training to carry out a caring task.

The immediate participant feedback questionnaires were positive (response rate 891/902, 99%), suggesting that the training was relevant (33% agreeing, 61% strongly agreed) and provided new information (47% agreeing and 43% strongly agreed). However, the response rate to a single posting (without reminders) of a 3-month questionnaire was very low (132/902, 15%); 89% of the staff who did respond reported finding the training materials useful. Staff reported that the training sensitised people to be aware of low mood and depression, and changed people’s perceptions of what to look for in a resident. Most respondents said they would have liked more training. One manager commented:

I think it was an eye-opener. There was the DVD she brought with her as well. I think it did get people questioning and there was a lot of interacting going on in that session (20 Manager BL).

#### Activity sweeps

Any shift in culture should have shown in changes in the levels and types of activities the residents were involved with. The best evidence we have for whether this happened comes from the activity sweeps, which we carried out in the eight case study homes. We carried out 109 activity sweeps; 53 at baseline and 56 at follow-up with 12 to 14 observations in each home at each timepoint. Table 1 shows the proportion of time spent in recreational activity, passive social interaction and being socially inactive varied considerably across the homes, but was remarkably consistent within each home between baseline and follow-up, suggesting that there was little or no observable change, at least in the eight case study homes (six of which were intervention homes). Residents spent a substantial amount of time in activities associated with eating and drinking, while active social interaction only amounted to between 5% and 15% of daily activity (Table 1). In more than 72 separate visits to case study homes (amounting to over 500 hours of observation), we observed only 1 exercise activity additional to that provided by OPERA in 1 home.

**Table 1:** Summary of daytime activity in case study homes, baseline versus follow-up

**Behaviour category codes (BCC)**[18,19]	**Timepoint**	**Home**^**a**^							
		**Intervention, % change**						**Control, % change**	
		**1**	**3**	**4**	**6**	**7**	**8**	**2**	**5**
Active social interaction	Baseline	4	5	14	7	9	10	9	15
	Follow-up	4	5	13	8	9	11	12	25
	Difference	0	0	−1	1	0	1	3	10
Eating/drinking	Baseline	22	22	17	20	29	21	23	27
	Follow-up	23	22	17	30	35	29	25	28
	Difference	1	0	0	10	6	8	2	1
Recreational activity (not exercise)	Baseline	15	22	23	27	30	15	20	31
	Follow-up	16	37	33	17	23	13	19	30
	Difference	1	15	10	−10	−7	−2	−1	−1
Exercise	Baseline	0	0	0	0	0	6	0	0
	Follow-up	0	0	0	0	0	5	0	0
	Difference	0	0	0	0	0	−1	0	0
Passive social interaction	Baseline	27	28	28	14	12	28	27	13
	Follow-up	32	16	14	14	17	25	25	11
	Difference	5	−12	−14	0	5	−3	−2	−2
Socially inactive	Baseline	29	14	16	29	17	15	15	11
	Follow-up	21	12	22	28	13	11	16	5
	Difference	−8	−2	6	−1	−4	−4	1	−6
Walking/wandering	Baseline	4	9	2	3	3	5	7	2
	Follow-up	4	8	1	3	3	6	3	1
	Difference	0	−1	−1	0	0	1	−4	−1

^a^Data based on between 12 and 14 observation sweeps of 90 minutes each covering the period from 10.30 AM in the morning to 17.30 PM in each of the 8 case study homes and is a mean percentage of the sweeps. Change is follow-up minus baseline.

During interviews, one manager noted the challenges faced in trying to increase mobility within the home, suggesting a culture that discourages increased activity and mobility.

It is a collective protective culture and that’s why they come into care. They [the care home staff and relatives] think people should be protected, but I think in this situation because we deal with people who have very high dependency needs that it sometimes is that we de-skill them, rather than enhance them because it’s a protective mechanism (44 Manager FU).

In summary, there was little observable evidence of a change in the culture in any of the case study homes including the six intervention homes, and time pressures on staff and reluctance of the management of some homes to put resources into supporting such a change might explain this lack of change, at least in part.

### Did the residents engage with the exercise?

#### Delivery of exercise classes

A total of 35 homes were randomised to the intervention, but after randomisation 1 home was found to be unsuitable for the intervention due to the extreme frailty of the residents and so no exercise groups were delivered in that home. We intended the 34 intervention homes to receive exercise classes at least twice a week for the year. We calculated that, allowing for delays in setting up classes and breaks due to public holidays, the total number of classes in each home would be around 92. The trial reached on average 90% of this target. The main reasons for cancelled classes were heavy snowfalls, outbreaks of diarrhoea and vomiting, swine flu precautions and high summer temperatures.

#### Attendance at exercise classes

The OPERA physiotherapists delivered a total of 3,191 exercise groups across the 34 intervention homes. These homes had a resident population of 1,439, of whom 494 (34%) were study participants. The physiotherapists assessed 16 residents (3%) as not being fit enough to participate in the exercise classes, so there were 478 study participants who were eligible to attend classes (Table 2). The average attendance at classes was ten residents, of whom, on average, five were study participants.

**Table 2:** Number of exercise groups, total attendances and reasons for non-attendance

**Category**	**Measure**	**Total study participants**	**Home populations**^**a**^
Residents (all)	n	494	1,439
Residents eligible to attend groups^b^	n (%)	478 (97)	1,256 (87)
Number of groups delivered	n	3,191	3,191
Number of groups available^c^	n	31,330	65,196
Total attendances^d^	n (%)	16,986 (54)	31,705 (49)
Average attendance	n	5.3	9.94
Groups attended:			
No groups	n (%)	43 (9)	187 (15)
51 or more^e^	n (%)	173 (36)	282 (22)
Reasons for non-attendance:			
Out	n (%)	372 (1)	1,449 (1)
In hospital	n (%)	719 (2)	1,656 (3)
Unwell	n (%)	1,670 (5)	3,167 (5)
Visitors	n (%)	369 (1)	765 (1)
Unwilling	n (%)	8,339 (27)	20,269 (31)
Other	n (%)	2,865 (9)	6,185 (9)

^a^Total home population including study participants and non-study participants.

^b^Eligible residents are those assessed as able to participate in the group exercise session (by the physiotherapist).

^c^Based on the number of groups eligible residents could have attended.

^d^Total number of person attendances at the available groups.

^e^Persons attending at least 51 groups (1 group per week or more during the lifetime of the trial).

In all, 9% of eligible participants did not attend any classes; around 20% attended 10 classes or fewer, while 36% attended 51 groups or more (at least 1 group per week). There was little attrition in the attendance at classes over the year (data not shown). An examination of group attendance by type of home revealed no differences (data not shown). Unwillingness to attend was the predominate reason for non-attendance (31%). Non-attendance was defined as refusal or other reason (including absence from home) for failure to attend a session that the resident was eligible to attend (Table 2).

When we analysed attendance at classes by characteristics of the study participants, we found no difference in attendance by age and SPPB. We found a significant difference between those who were depressed at baseline compared to those not depressed (*P* <0.001) with those with depression on average attending fewer groups than those who were not depressed (median values 21 and 43, respectively). Interestingly we also found a difference between those with high and low levels of cognitive impairment (MMSE), with those with lower MMSE scores attending significantly more groups (median values 35.5 and 29.5 respectively, *P* = 0.009) (Table 3).

**Table 3:** Average exercise group attendances of study participants (N = 494) grouped by baseline variables

**Grouping**						
	**n**	**Mean**	**SD**	**Median**	**Interquartile range**	***P *** **value**
Grouped by age^a^						0.673^b^
Below and equal to mean	224	35.38	27.91	34.00	6.00 to 60.00	
Above mean	263	33.89	28.24	31.00	6.00 to 59.00	
Grouped by MMSE^c^						0.009^b^
Below and equal to median	208	38.41	26.86	35.50	13.00 to 62.00	
Above median	228	33.31	28.51)	29.50	2.25 to 59.00	
Grouped by SPPB^d^						0.160^e^
Score of 0	160	32.11	27.55	28.00	6.00 to 57.75	
Score between 1 to 2	136	37.56	27.92	37.50	10.00 to 62.00	
Score between 3 to 4	77	39.78	29.34	48.00	9.00 to 67.50	
Score 5+	57	35.54	25.99)	42.00	6.50 to 56.50	
Grouped by depression^f^						<0.001^b^
Not depressed	228	40.30	28.33	43.00	12.25 to 66.00	
Depressed	193	29.88	26.26	21.00	3.50 to 54.00	

^a^Split into two age groups based on mean (86.94 years).

^b^Independent samples Mann–Whitney U test.

^c^Mini Mental State Examination (MMSE). Split into two groups based on median (19).

^d^Short Physical Performance Battery (SPPB). Split into four groups to capture the high number of 0 scores.

^e^Independent samples Kruskal-Wallis test.

^f^Split into two groups combining those with moderate and severe depression (depressed) against those with ‘none’.

#### Intensity of exercise intervention

The exercise intervention was designed to facilitate three levels of participation, from all seated to all standing exercises, but the majority of sessions were predominantly seated (level 1) due to the frailty of the participants. The study sample were predominantly female (76%), white (98%) with a mean age of 86 years (± 7.3 years). Baseline SPPB scores, a measure of physical function, were very low in intervention home participants (mean 1.9 ±2.2), as were the baseline MMSE scores (mean 18.7 ±6.9).

#### Fidelity of the exercise intervention

Fidelity was low for the whole home approach, with only 56% of the physiotherapists judged to be satisfactorily achieving delivery of the whole home approach (Table 4). Intervention delivery by the physiotherapist was judged to be fairly good, with 80% achieving a satisfactory level. Groups observed by DRE were delivered as protocol and were well received by the residents; however, it was clear, the physiotherapists found it difficult to spend extra time in the homes to encourage whole home activities. Care staff were often too busy to support the physiotherapists getting residents to and from groups and indeed helping during groups, limiting opportunities for staff/physiotherapist interactions.

**Table 4:** Quality assurance observation tool, with percentage achieved during visits

**Item**	**Not achieved, n (% of total)**	**Partially achieved, n (% of total)**	**Satisfactorily achieved, n (% of total)**
Administration/record keeping (completion of all forms/registers)	3 (7)	8 (20)	30 (73)
Preparation for group (room, residents, resources, staffing)	0 (0)	0 (0)	41 (100)
Storage and organisation of equipment	0 (0)	1 (3)	40 (97)
Personal performance in running group (use of communication, music, facilitation)	0 (0)	6 (15)	35 (85)
Content of group exercise session (intensity, progression, equipment use)	0 (0)	8 (20)	33 (80)
Whole home approach activities/communication/cooperation/support to care staff, mobility recommendations, equipment procurement	0 (0)	18 (44)	23 (56)

#### Staff and residents’ views of exercise intervention

Despite the relatively low attendance at classes, staff and residents were positive about them, and staff also appreciated having a professional in charge of the exercise class.

You know, at first even when I used to do my little 5 minutes and workouts or we’d do a daily dance, it could be quite worrying; ‘Oh my legs hurt so I’m not going to do anything’, whereas because they’ve got a professional guiding them, using the weights and even telling her what weights to use… You know, there’s a sudden interest in their own wellbeing (27 Activities Coordinator).

The positive impact of the exercise groups was mentioned by many interviewees, talking about changes in residents’ mood and physical ability about the changes they have seen in residents’ attitude towards exercise:

I’d often walk by and see them and they were always smiling. There was always…there was a good rapport in there. There was always laughter in there. The music would be going and you would see them all doing this [gesture] and I never saw anybody sitting there sort of looking glum (32 Senior Carer).

#### Observation of exercise intervention

The challenges faced by the OPERA physiotherapists were evident in the observation of the classes. Managing a group with a range of levels of ability could be exacting and some of the residents had challenging behaviour. Nevertheless, the pleasure the residents took in the classes was obvious, with laughter and smiles and residents singing along to the music.

## Discussion

Few trials have included such a comprehensive process evaluation carried out independently of the main trial. The OPERA process evaluation has given us a unique insight into both workings of the care home sector for older residents and the implementation of a large complex cluster randomised controlled trial in this setting. It is important to try and understand why an intervention is effective or not. Our study is one of a growing number of published randomised controlled trial process evaluations [22-26]. Here, we explore two possible explanations for the negative result of the OPERA trial: whether the intervention changed the culture of the homes to promote more physical activity among residents, and whether the residents engaged with the exercise classes.

### Did the intervention change the culture in the homes?

One of the most striking findings of this evaluation is the difference between care homes within the OPERA study. However, all the homes in OPERA volunteered to be in the study so we might imagine that the differences across the entire care home sector would be even more extreme. The activity sweeps show patterns of activity that appear to be relatively consistent across time within the homes but very different between homes. Differences in the mix of residents alone seem unlikely to explain why the culture around physical activity appears to differ between homes. It is unclear how the very different physical environments we observed might impact on the residents and care staff and further work exploring these environmental issues is ongoing [27]. The process evaluation shows that it was possible to run standardised, evidence-based exercise groups requiring special equipment in all the homes in the OPERA study. The stability of patterns of activity across 12 months within the case study homes suggests that changing the culture of homes, at least with regard to patterns of activity among residents, might be more difficult.

While the physiotherapists developed a very good relationship with the homes and residents, the planned provision of a ‘home champion’ did not happen. Possibly the best placed member of staff to take on this role would have been the activities coordinator. However, while a number of activity coordinators were observed just fulfilling this role many were also allocated other tasks, taking them away from the ‘activity’ role. Where the role was recognised and exclusive residents benefited from having someone who was dedicated to increasing activity, but this was rare and in general the identification of ‘home champions’ was not successful. It seems that the role of home champion was unrealistically demanding for most staff in most homes.

Access to a health professional was something home managers and staff found beneficial. Most of the managers and staff interviewed reported problems with obtaining basic NHS services for residents such as physiotherapy, or even to obtain provision of simple mobility aids. The British Geriatrics Society has highlighted the issue of providing seamless healthcare provision for patients who are resident in homes in the independent sector [28]. Our process evaluation identified that OPERA physiotherapists succeeded in obtaining simple, relatively low cost mobility aids (from NHS sources) that care homes had been either unaware of, or unable to access.

The OPERA physiotherapists assessed residents and provided aids, equipment and advice which should have made it easier for homes to increase the activity of residents when the OPERA staff were not there. There was little evidence that this happened. Each resident had an individual exercise prescription yet there was little or no evidence that homes made use of these.

Care staff were positive about the staff training sessions, when they attended. However, it is doubtful if this one-off session with only just over half the staff attending was sufficient to bring about the necessary changes; particularly when the training promoted the safe mobility of residents as a mood enhancer.

The OPERA study took place across a period of increasing economic uncertainty and, latterly, in an evolving economic recession. The majority of UK care homes are part of the independent sector and one of the largest groups in the UK, Southern Cross, announced it was closing down just after the end of the study [29]. Even at baseline, mean occupancy was 87% and the field team reported that vacant places increased across the duration of the study. Observation and interviews in the homes suggested that care staff often have to work very hard and some resource-stretched homes may have little spare capacity to engage in cultural shifts that consume carer time, such as promoting physical activity among frail, older residents.

### Did the residents engage with the exercise?

The exercise groups were delivered as planned and information from the focus groups and interviews identified many positive effects of the groups and almost universal enthusiasm from the home staff and residents for the groups, which only appeared to increase across the duration of the study. Moreover there was little evidence of fall-off in the numbers attending of the classes across time, suggesting that an ongoing exercise-class-based intervention in care home settings is viable.

On the surface it seems that attendances at group sessions were good, with some attending every session, but a closer look at the data reveal a number of issues. There were a large number of residents coded as ‘unwilling’ to attend. It is difficult to know if this was real unwillingness or because staff or the physiotherapists did not have time to gather residents together for the session. The physiotherapists reported that, when they arrived for a group session, the care staff were often too busy to help get people to the group. Less than half of study participants (that is, those who may have contributed to the primary outcome) attended an average of one session per week (the predefined definition of an ‘adequate dose’) or more. Thus, overall exposure to the group sessions was poor.

The frailty of the residents and high levels of cognitive impairment may be a contributing factor. The SPPB results revealed that many residents had poor physical function (particularly in the lower limbs), contributing to the fact that the exercise groups were mostly seated. Residents enjoyed the sessions and exhibited outward signs of joy and happiness. However, perhaps they did not reach an intensity of physical activity sufficient to bring about long-term physiological or psychological mood enhancing changes.

Secondary analysis of the impact of a number of baseline variables on group attendance reveals that neither age, poor physical function (SPPB) nor greater cognitive impairment (MMSE) seemed to stop residents attending the groups. What we did find is that those who were not depressed attended significantly more groups than those who were depressed. This suggests that our exercise intervention was missing those who may have benefited most from it. In future, it may be useful to explore how these depressed residents could be encouraged to join in the group activities. We also found that those with lower MMSE scores attended more groups, which is somewhat counterintuitive. What it does suggest is that cognition is not a barrier to attendance at groups such as these but we are cautious at overinterpreting this result that is based on a *post hoc* analysis.

Two other similar but smaller trials have tested interventions to increase physical activity in older people with depressive symptoms. Neither of them found any effect of the intervention on the prevalence of depressive symptoms. One of these trials was delivered to older people in residential care but did not report any process data that would allow further exploration of possible reasons for the negative result [30]. A trial with community-dwelling older people tested individually prescribed moderate intensity exercise combined with walking, each for 30 minutes three times a week [31]. The researchers reported good uptake in the intervention group, with 84% of randomised individuals receiving all the planned visits, although the participation in the prescribed activity was less. At 6 months just 29% were carrying out the exercises three times a week, and 37% were walking three times a week. Another trial of physical activity in older people provided detailed process evaluation of a negative result, but was not targeted at reducing depression but on reducing falls [22]. The researchers tested providing t’ai chi classes twice a week for 13 weeks. Both instructors and participants were very positive about the classes, but nevertheless, only 64% of the interventions group completed the course and only 47% were rated as having achieved an adequate attendance of at least 21 of the 26 classes. It seems that low uptake and adherence may be a common issue although neither of the two trials reporting uptake took place in a residential facility. In addition, the fact that the participants were living independently implies that they were on average considerably less frail than the participants in the OPERA trial. A recent Cochrane review of exercise for depression found that exercise may have a positive effect on depression, but there is considerable uncertainty about the optimum duration and frequency of the exercise interventions to achieve a positive outcome [32]. Few of the trials included frail care home residents similar to those in the OPERA trial; as noted earlier, most were less frail and more mobile.

This process evaluation had some limitations. The number and spread of interviews carried out was smaller than planned, particularly with regard to home residents. Relatives were also difficult to access as we had no access to their contact information. Our detailed observations were limited to 8 of the 78 homes in the trial. Nevertheless, even in this small sample we observed a wide diversity in levels of activity within the homes. We are cautious in our interpretation of the *post hoc* analyses, as these were unplanned, unadjusted and lack power. We have therefore provided a full set of descriptive statistics to enable the reader to draw their own conclusions.

We have explored in detail two possible reasons why the OPERA trial was negative. Eliciting a cultural shift in the care home sector is problematic and the OPERA intervention failed to bring about enough of a change. We have also found that the care home population is older and frailer than anticipated when the OPERA trial was planned, perhaps as a result of a prevailing ethical and financial imperative to maintain frail older people in their own homes with support for as long as possible. While the staff and residents were positive about the exercise groups it appears that the intervention did not encourage enough engagement or intensity of exercise to address low mood more than momentarily, and that those with most potential to benefit from an intervention ameliorating depression (that is, the depressed) were least likely to engage in it. Indeed, from many hours observing the intervention, we appeared to make a difference. These differences may have just been transient or, alternatively, the measures we were using may not have been sensitive enough to capture them. It is clear that life for our aging population in a care home setting can be very sedentary and this increases the possibility of low mood and a poor quality of life. Alternative approaches are needed to both relieve the burden of depression in care home residents and to improve their overall quality of life. Researchers in the future need to consider that the instruments we use to measure depression and quality of life may need to be tailored to the population being studied, and any promising approaches should be tested empirically before implementation.

## Conclusions

In summary, it remains possible that increasing physical activity could reduce depression among care home residents but the OPERA pragmatic RCT and its process evaluation suggests that it may not be feasible to design and deliver an intervention that would achieve the necessary level of increased physical activity in a real-world setting.

## Competing interests

The authors declare that they have no competing interests.

## Authors’ contributions

MU was chief investigator of the OPERA trial. DRE, MT, SJCT and MU were involved in conception and design of the process evaluation. DRE collected and managed the data overseen by MT and SJCT. DRE and CS were responsible for data analysis. DRE drafted the manuscript supported by all authors. All authors contributed to interpretation of results, and drafting of the final article. All authors read and approved the final manuscript.

## Pre-publication history

The pre-publication history for this paper can be accessed here:

http://www.biomedcentral.com/1741-7015/12/1/prepub
